# High Efficient and Environment Friendly Plasma-Enhanced Synthesis of Al_2_O_3_-Coated LiNi_1/3_Co_1/3_Mn_1/3_O_2_ With Excellent Electrochemical Performance

**DOI:** 10.3389/fchem.2020.00072

**Published:** 2020-03-20

**Authors:** Xinzhi Wang, Qianqian Jiang, Yichi Zhang, Nannan Yuan, Jianguo Tang

**Affiliations:** ^1^Institute of Hybrid Materials, National Center of International Research for Hybrid Materials Technology, National Base of International Science and Technology Cooperation, College of Materials Science and Engineering, Qingdao University, Qingdao, China; ^2^School of Chemical and Biomedical Engineering, Nanyang Technological University, Singapore, Singapore

**Keywords:** plasma-enhanced method, Al_2_O_3_ layer, LNCM, elevated temperature performance, Li-ion batteries

## Abstract

PLA-1-Al_2_O_3_@LNCM synthesized using an efficient and facile plasma-enhanced method exhibits markedly improved capacity retention of 98.6% after 100 cycles, which is much larger than that of LNCM at 80% after 100 cycles. What is more, it also exhibits significantly enhanced cyclicity compared to that of 1-Al_2_O_3_@LNCM cathodes prepared using the normal solid state method, which further illustrates the efficiency and superiority of this plasma-enhanced method. More importantly, the rate performance of PLA-1-Al_2_O_3_@LNCM is improved because of the better electrolyte storage of the assembled hierarchical architecture of the Al_2_O_3_ coating layer according to unimpeded Li+ diffusion from electrode to electrolyte. When cycling at 55°C, the PLA-1-Al_2_O_3_@LNCM shows 93.6% capacity retention after 100 cycles, which is greatly enhanced due to the uniform Al_2_O_3_ layer. Further, growth of polarization impedance during cycling can be effectively suppressed by the Al_2_O_3_ layer, which can further confirm the effect the Al_2_O_3_ layer coated on the surface of the LNCM. The enhanced cycling performance and thermal stability illustrates that this facile surface modification, using the plasma-enhanced method, can form an effective structured coating layer, which indicates its prospects as an application in the modification of other electrode materials.

## Introduction

With the development of the global energy demand and the urgency of protecting the environment, there is a growing need to develop clean and more efficient power sources like lithium ion batteries (LIBs) (Tang et al., 2013; Chen et al., 2016), which have become the most feasible power source for portable electronic devices, mainly due to their high gravimetric and volumetric energy densities (Xu et al., 2017; Lv et al., 2019) Considering that the specific capacity of LIBs heavily relies on cathode materials, efforts have been made to develop cathode materials with a high energy density and large power capability (Lee et al., 2014; Zhang et al., 2018).

Until now, in cathode materials, such as layered LiCoO_2_ (Zhao et al., 2015), spinel LiMn_2_O_4_ (Jiang et al., 2016b), LiNiO_2_, and olivine LiFePO_4_ have been widely applied for commercial LIBs in electrical vehicles (EVs) or hybrid electric vehicles (HEVs). Among them, LiCoO_2_ is the earliest commercialized cathode material which cannot meet the increasing practical demand due to its low practical capacity, relatively high cost, toxicity of cobalt, and unreliable safety. Researchers have therefore focused on finding alternative cathode materials to satisfy the required environmental, safety, and cost standards for LIBs (Ohzuku and Makimura, 2001; Deng et al., 2008; Makkus et al., 2010). In comparison with LiCoO_2_, layered LiNi_x_Co_y_Mn_z_O_2_ (x + y + z = 1) is considered an excellent candidate as a cathode for commercial LIBs. In 2001, LiNi_1/3_Co_1/3_Mn_1/3_O_2_ (LNCM) was successfully implemented, and has the same layered α-NaFeO_2_ structure with overall hexagonal symmetry (space group R-3 m) as LiCoO_2_ (Xia et al., 2014) but with a much higher capacity, better thermal stability, is more environmental friendly and costs less (Lee et al., 2004; Luo et al., 2006; Cho et al., 2011). Nevertheless, LNCM still has some disadvantages, such as an inferior cycle performance at high working voltage, relatively poor conductivity, and irreversible side reactions with electrolytes and cation a disorder between Li^+^ and Ni^2+^, which leads to some technical challenges related to cyclability and rate capability, seriously affecting its large-scale applications (Yao et al., 2013; Cui and Xu, 2015; Zhu et al., 2015). Researchers worldwide have proposed various solutions to overcome these disadvantages. For example, surface coating is an easy and effective way to improve the electrochemical performances of cathodes. Various coating materials such as carbon and metal oxides (Al_2_O_3_, Li et al., 2014; Li_2_TiO_3_, Lu et al., 2013; ZrO_2_, Hu et al., 2009; TiO_2_, Li et al., 2006; AlF_3_, Myung et al., 2010; AlPO_4_, Wang et al., 2013, etc.) have been studied, which can increase the electronic conductivity and inhibit the side reactions between cathode and electrolyte (Jiang et al., 2018). Among the metal oxides mentioned above, Al_2_O_3_ is regarded as a desirable surface coating material due to its excellent chemical and thermal stability, which is good for the electrochemical performance cathode material. However, the normal method used for coating Al_2_O_3_ is solid-state (Kim et al., 2006) or solution methods (Qiu et al., 2014), which often result in no uniform elemental distribution and inferior electrochemical performance (Yan et al., 2016). In addition, the experimental conditions (pH, temperature, and mixing speed) should be carefully controlled to synthesize homogeneous hydroxide or carbonate precipitates, making this method complicated (Manthiram et al., 2016). Therefore, it becomes challenging to develop a facile and general approach for the preparation of coating on the LNCM with favorable structures and excellent electrochemical performance.

**Scheme 1:**
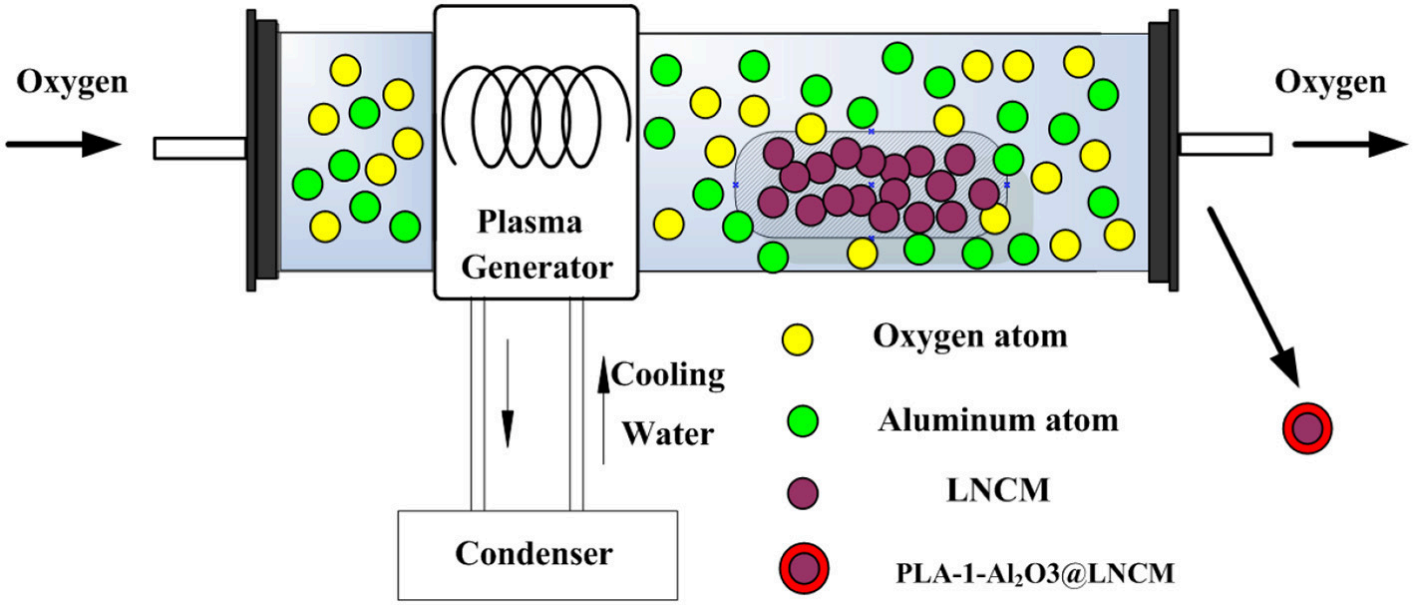
Schematic diagrams for PLA-1-Al_2_O_3_@LNCM.

Herein, we first report a novel facile plasma-enhanced low-temperature approach in LiNi_1/3_Co_1/3_Mn_1/3_O_2_ coated with Al_2_O_3_ with a superior hierarchical structure, as illustrated in Scheme 1. This method produces a simple, uniform coating, which can delay the cathode/electrolyte interfacial reactions and facilitate Li-ion diffusion in the batteries. The cell made of LiNi_1/3_Co_1/3_Mn_1/3_O_2_ coated with Al_2_O_3_ delivers a high specific reversible capacity of 213.8 (0.2°C), 208.4 (0.5°C), 199.6 (1°C), and 189.0 (2°C) mAhŁEg^−1^, respectively. More importantly, all the LiNi_1/3_Co_1/3_Mn_1/3_O_2_ coated with Al_2_O_3_ composite electrodes show improved cycling performance and rate capability, especially in an elevated temperature, compared to the pristine LiNi_1/3_Co_1/3_Mn_1/3_O_2_ electrode, which further illustrates the effect of the Al_2_O_3_ layer as a medium of preventing the material from degradation during cycling.

## Experimental

### Synthesis of PLA-1-Al_2_O_3_@LNCM and 1-Al_2_O_3_@LNCM Cathode Electrodes

The LiNi_1/3_Co_1/3_Mn_1/3_O_2_ coated with Al_2_O_3_ layer was prepared by an efficient plasma-enhanced method. In order to make the uniform mixture, the LiNi_1/3_Co_1/3_Mn_1/3_O_2_ (denoted as LNCM) powder was supplied by one Tech. Company. Aluminum isopropoxide (C_9_H_21_AlO_3_, purchased from Aladdin official online mall) was chosen as the source of aluminum, and the Al content was set at molar ratios of Al:(Ni + Co + Mn) = 0, 0.5, 1.0, and 1.5% by controlling the amount of C_9_H_21_AlO_3_. First, the mixture was transferred into the plasma-enhanced tube furnace. The oxygen gas was introduced into the reactor for about 20 min to remove the air in the tube. The temperature of the tube furnace was set to 350°C with the heating rate at 10°/min. Then the pressure was controlled under 50 Pa. During the process, the applied RF power was set to 200 W. The plasma treatment time was controlled for 20 min. The samples of LiNi_1/3_Co_1/3_Mn_1/3_O_2_, coated with Al_2_O_3_ synthesized by the plasma-enhanced method using C_9_H_21_AlO_3_ as the Al source, was defined as PLA-X Al_2_O_3_@ LNCM (X represents the Al content: 0.5, 1.0, and 1.5%). For comparison, the conventional annealing method was also utilized to prepare LiNi_1/3_Co_1/3_Mn_1/3_O_2_ coated with a Al_2_O_3_ layer using C_9_H_21_AlO_3_ for the Al source. The mixed powders with an Al content of 1% were sintered at 500°C for 6 h. Finally, the Al_2_O_3_-coated LiNi_1/3_Co_1/3_Mn_1/3_O_2_ was obtained after naturall cooling the product to room temperature (denoted as 1-Al_2_O_3_@LNCM).

### Materials Characterizations

The crystalline phases and morphologies of the samples were characterized by an X-ray diffractometer (D/MAX-2200V, Rigaku, Japan) using Cu Kα radiation (k = 0.15406 nm) and scanning electron microscopy (JEOL JSM-7800F, Japan) equipped with an energy dispersive spectrometer (EDS) for elemental analysis. X-ray diffraction studies were carried out using Cu-Kα radiation. The morphology, crystal lattice, and the micro-region structure of the sample coating layer were characterized by the HR-TEM (HR-TEM, JEOL JEM-2100Plus, Japan).

### Cell Fabrication and Electrochemical Measurements

Electrochemical experiments performed in R2032 coin type cells were assembled in an argon-filled glove box, using lithium metal as the counter electrode. The electrolyte consisted of a solution of 1 M LiPF_6_ in ethylene carbonate (EC), dimethyl carbonate (DMC), and ethyl methyl carbonate (EMC) solution (1:1:1 by volume). The composite electrodes were made from the active materials powder (80 wt.%), acetylene black (10 wt.%), and polyvinylidene fluoride (PVDF) binder (10 wt.%), homogeneously mixed in Nmethyl pyrrolidinone (NMP) solvent. The slurry was then coated onto the aluminum foil current collector and dried under a vacuum at 100°C for 12 h. The mixed slurry was pasted on Al foil and subsequently dried at 120°C for 12 h under a vacuum and then roll-pressed. All cells were assembled in an argon filled gloved box. The galvanostatic charge and discharge cycle tests were carried out at 25 and 55°C between 2.8 and 4.3 V using a battery tester at the current density of 0.2°C (1°C = 1,675 mAhŁEg^−1^) (LAND CT2001A, China). The electrochemical impedance spectra (EIS) were conducted on UT85794 (made in the Netherland) in the range from 100 kHz to 10 mHz.

## Results and Discussion

**Figure 1:**
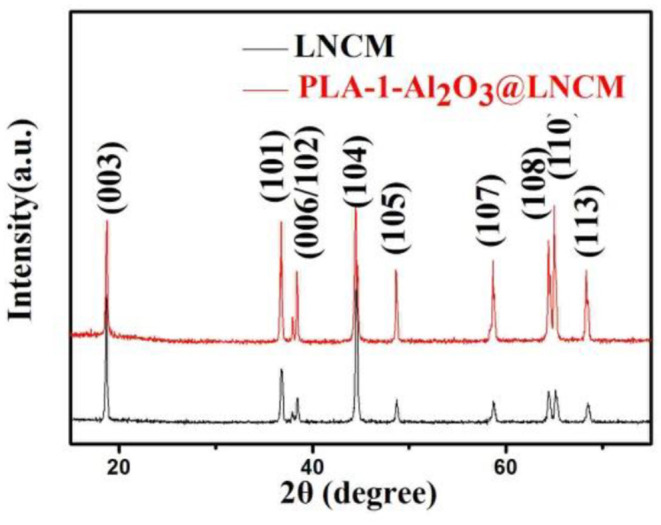
Powder X-ray diffraction patterns of LNCM and PLA-1-Al_2_O_3_@LNCM.

The X-ray diffraction patterns of the bare LNCM and PLA-1- Al_2_O_3_@LNCM are shown in Figure 1. All the peaks of the two samples in the XRD patterns can be indexed to the NaFeO_2_ structure using space group R-3m without impurity phases, and matched well with those reported in the literature (Li et al., 2018). Furthermore, distinct splitting (006)/(102) and (108)/(110) peaks were observed in all the XRD patterns, indicating that a well-formed layered structure was obtained, illustrating that the thin Al_2_O_3_ surface coating did not change the structure of the LNCM. No peak of Al_2_O_3_ was observed due to the low quantity, which reveals that only Al_2_O_3_ exists and no other impurity presents during the plasma-enhanced coating process. To confirm the differences between PLA-1-Al_2_O_3_@LNCM and LNCM, the lattice parameters calculated by Rietveld refinement programs are shown in Table S1. From this data, it can be seen that a slight difference is noted for PLA-1-Al_2_O_3_@LNCM, which has a slight increase in the a and c axes. In addition, there is no shift in the peaks, indicating that Al-doping does not take place during the plasma-enhanced process, further illustrating the efficiency of the plasma-enhanced method for the preparation of PLA-1-Al_2_O_3_@LNCM electrode materials.

**Figure 2:**
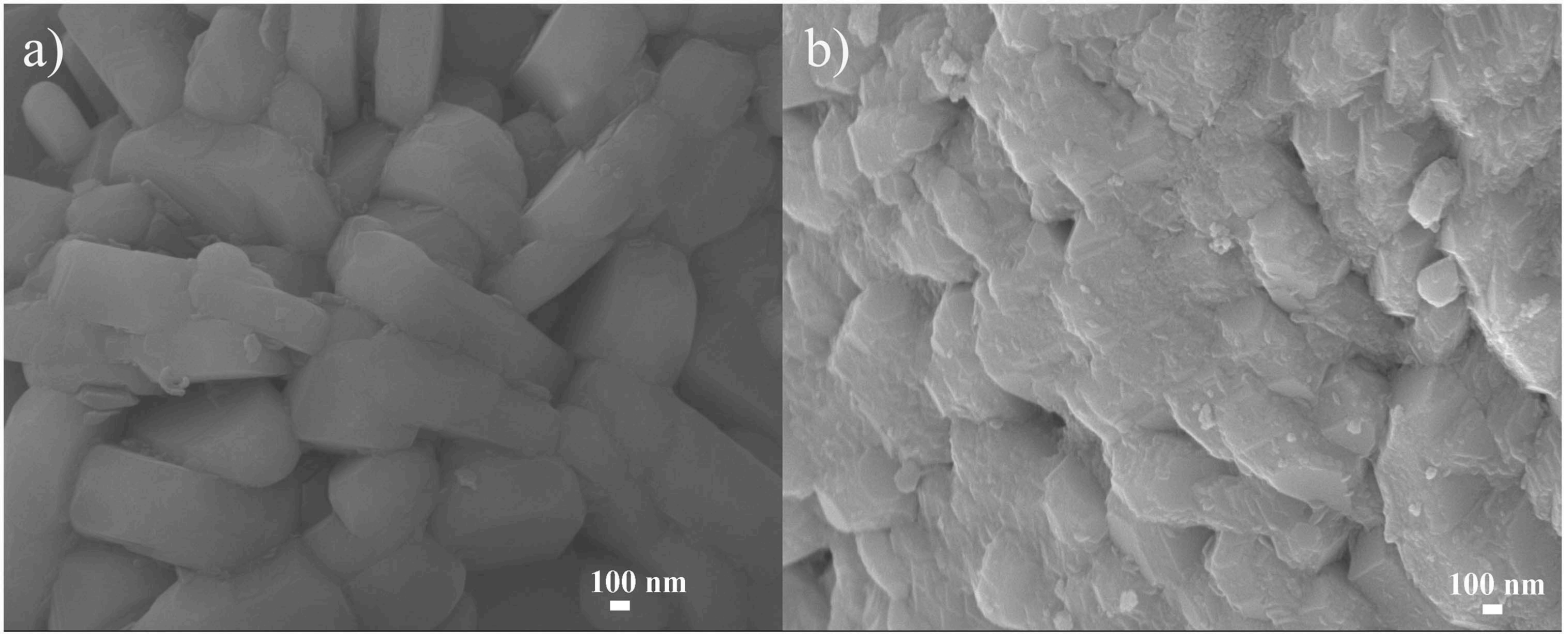
SEM images of **(a)** LNCM and **(b)** PLA-1-Al_2_O_3_@LNCM.

The morphologies of LNCM and Al_2_O_3_ coated LNCM are shown in Figure 2. The two samples fall under a similar size range of 0.5–0.8 μm. It can however, clearly be seen that the surface of PLA-1-Al_2_O_3_@LNCM becomes rough and loses its smooth contours, due to the Al_2_O_3_ grown on the surface of LNCM with layermorphology. When analyzed using EDS during SEM analysis, as-prepared Al_2_O_3_ coated electrodes, clearly revealed that the molar ratios of Ni, Co, and Mn of the two samples are both close to 1:1:1, which agrees with the ideal layer structure LNCM. In addition, it exhibits an increase in Al and O content compared to that of pristine LNCM as shown in Figure S1, which can further confirm that the Al_2_O_3_ has successfully grown on the surface of LNCM. At the same time, the SEM images of the LNCM with different Al_2_O_3_ consent, have been invested as shown in Figure S2. We can see that when the consent of Al_2_O_3_ is 1 wt.%, it shows a uniform morphology, which will be good for the electrochemical performance of the material. When the consent of Al_2_O_3_ is less or more, it may not grow uniformly or may aggregate on the surface of LNCM, which will hinder the speed of the lithium ion insert/disinsert during cycles. SEM images of the PLA-1-Al_2_O_3_@LNCM and LNCM, after cycling at 0.2°C, are shown in Figure S3. It can be clearly seen that the SEM image of PLA-1-Al_2_O_3_@LNCM presents no obvious changes in the morphology, which further illustrates the stability of sample PLA-1-Al_2_O_3_@LNCM due to the uniform Al_2_O_3_ layer.

**Figure 3:**
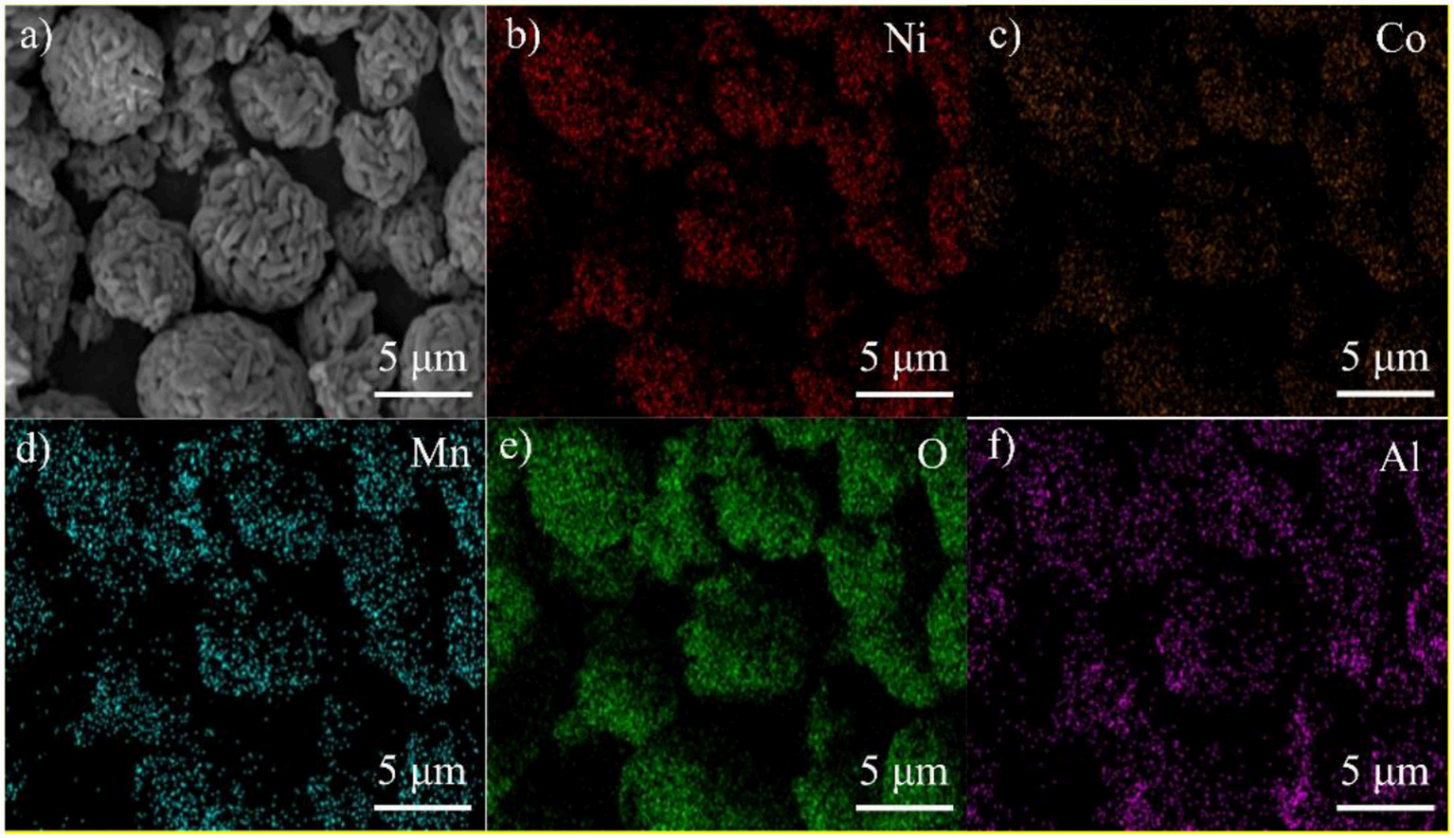
EDS maps for PLA-1-Al_2_O_3_@LNCM: **(a)** SEM image PLA-1-Al_2_O_3_@LNCM powders, corresponding elemental mappings images of **(b)** Ni, **(c)** Co, **(d)** Mn, **(e)** O, and **(f)** Al.

In order to check the uniformity of the Al_2_O_3_ coating layer on the surface of LNCM, EDS mapping was performed, as shown in Figure 3. The bright dots assigned to the elements O and Al are similar to the SEM of the PLA-1-Al_2_O_3_@LNCM sample, which indicates that Al_2_O_3_ has uniformly dispersed on the surface of LNCM. This result is consistent with that of XRD patterns, SEM, and EDAX, which further confirms that the Al_2_O_3_ layer has successfully grown on the surface of LNCM.

**Figure 4:**
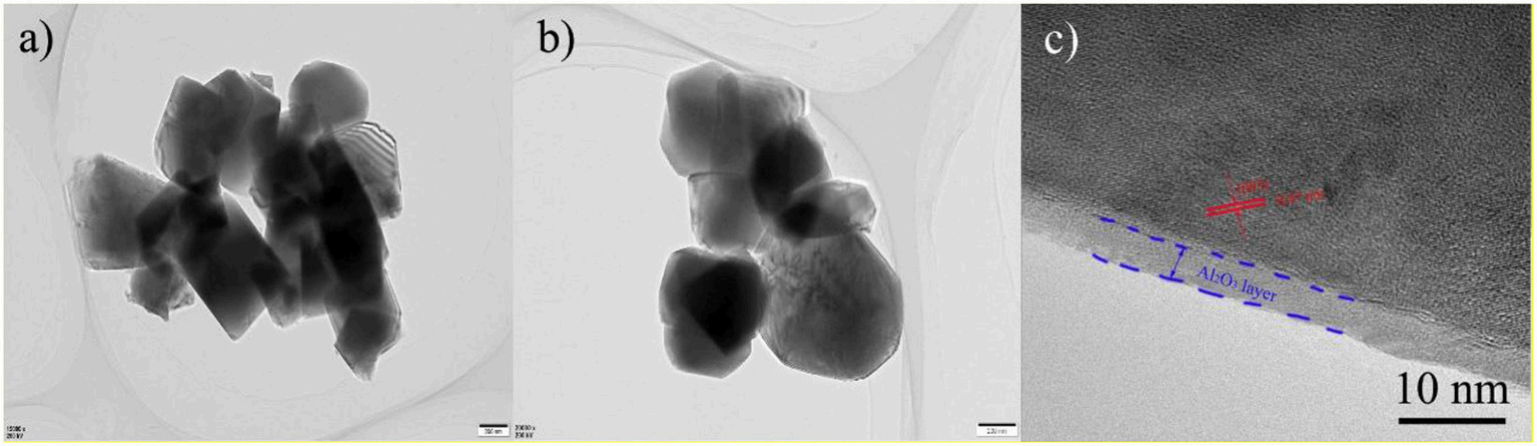
TEM image of **(a)** LNCM and **(b)** PLA-1-Al_2_O_3_@LNCM, **(c)** HR-TEM image PLA-1-Al_2_O_3_@LNCM.

In order to further analyze the thickness and microstructure of Al_2_O_3_ on the surface coating layer, TEM of LNCM and PLA-1-Al_2_O_3_@LNCM materials at different magnification are shown in Figure 4. Because the coating carbon layer is very thin, it is difficult to find any obvious differences in Figures 4a,b. However, the HR-TEM image in Figure 4c clearly shows that the host LNCM with interplanar distances of the parallel lattice value of 0.47 nm, indexed to the distance of the closely packed (003) plane of the R3m-layered structure (Guo et al., 2015), which illustrates that the Al atom has not changed the structure of LNCM and only grows on the surface of LNCM. In addition, it can be seen that the Al_2_O_3_ coating layer with an amorphous phase is about 5 nm, and is clearly distinguishable from the crystalline LNCM, which is consistent with XRD and SEM analysis. TEM analysis demonstrates that the coating method is an effective way to coat the Al_2_O_3_ layer on the surface of LNCM, which can reduce the contact area between the electrode and electrolyte, and further protects the cathode material from dissolving into the electrolyte.

**Figure 5:**
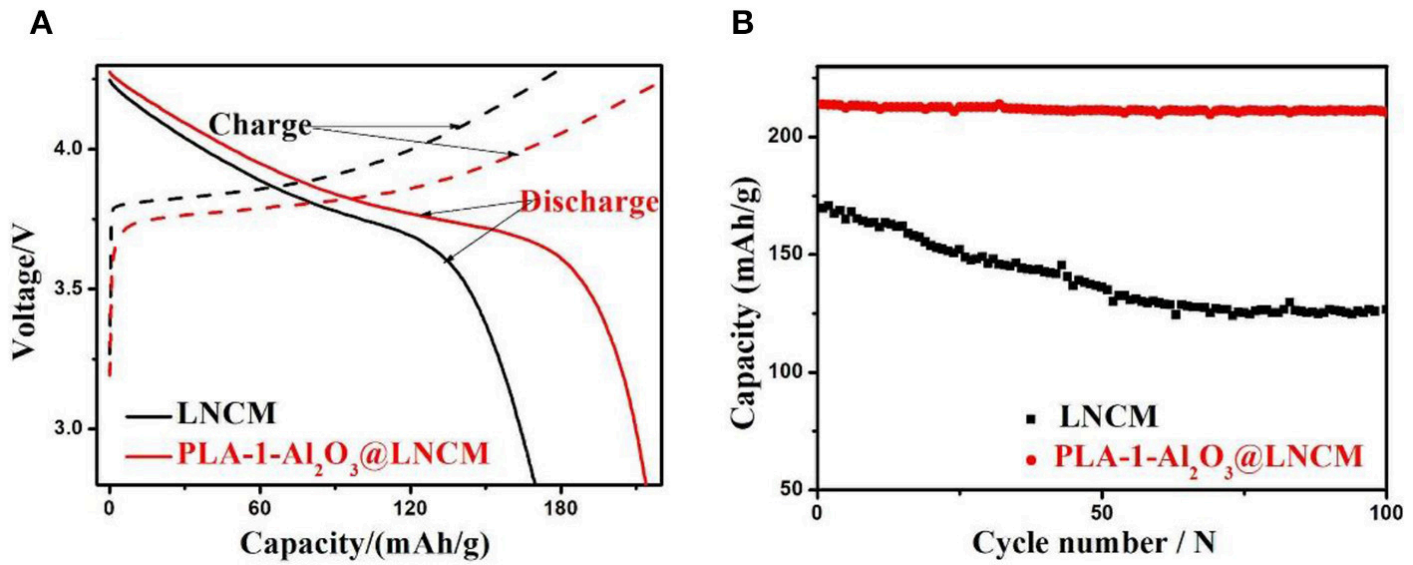
**(A)** The initial discharge curves and **(B)** Discharge capacities vs. cycle number of LNCM and PLA-1-Al_2_O_3_@LNCM at the rate of 0.2°C in the voltage of 2.8–4.3 V at room temperature.

The initial charge-discharge capacity of PLA-1-Al_2_O_3_@LNCM at the rate of 0.2°C in the voltage of 2.8–4.3 V at room temperature are compared with LNCM as shown in Figure 5A. It can be seen that the initial discharge capacity of PLA-1-Al_2_O_3_@LNCM is much higher than that of bare LNCM, probably due to the uniform Al_2_O_3_ layer, which can improve the electrical conductivity of the material and even prohibit the Jahn-Teller distortion occurring at the surface under non equilibrium conditions (Jiang et al., 2016a). At the same time, LNCM coated with different Al_2_O_3_ consent are also characterized in Li-ion batteries (as shown in Figure S4A). Among the three samples, PLA-1-Al_2_O_3_@LNCM shows the highest initial discharge performance, due to the suitable thickness of Al_2_O_3_ formed only with the consent Al of 1%. In order to further make sure the efficient of this plasma-enhanced method, the initial discharge performance of Al_2_O_3_@LNCM synthesized form the normal solid state method are invested as shown in Figure S5A. The initial discharge of Al_2_O_3_@LNCM is 196.9 mAh·g^−1^, which is lower than that of PLA-1-Al_2_O_3_@LNCM. This result illustrates this method not only can improve the efficient of this coated experiment, but also can improve the electrochemical performance of the material, indicating that the plasma-enhanced method can synthesized the coated LNCM with suitable Al_2_O_3_ layer with lower temperature and less reaction time.

Figure 5B summarizes cycling performances of LNCM and coated LNCM cathodes cycled at 0.2°C in a potential range of 2.8–4.3 V at room temperature. Capacity retention of PLA-1-Al_2_O_3_@LNCM upon cycling between 4.3 and 2.8 V is 98.46%, which is much higher than that of LNCM (74.66%). In other words, the discharge capacity of LNCM only maintained 74.66% of its initial capacity; while the discharge capacities of PLA-1-Al_2_O_3_@LNCM only decrease slightly from 213.8 to 210.5 mAhŁEg^−1^ after 100 cycles, maintaining 98.46% of its initial capacity, which may be associated with cobalt dissolution and structural deterioration at the high operating voltage, resulting in increased interfacial resistance. At the same time, LNCM coated with different Al_2_O_3_ consent are also characterized in Li-ion batteries as shown in Figure S4B. The capacity of The PLA-0.5-Al_2_O_3_@LNCM and PLA-1.5-Al_2_O_3_@LNCM samples suffers 11.51 and 10.54% capacity loss, respectively, while PLA-1-Al_2_O_3_@LNCM has the highest capacity retention of 98.46% after 100 cycles at 0.2°C between 2.8 and 4.3 V. It can be seen that when the mass concentration of Al_2_O_3_ is less or more than 1 wt.%, the capacity loss also undergoes a certain fading, which further illustrates that the suitable consent of Al content is 1 wt.%. For the PLA-1-Al_2_O_3_@LNCM sample with a proper coating amount, the hierarchical nanosheets of metal oxide can sequester HF in electrolytes containing LiPF_6_. The rich pores improve the ionic conductivity of the coating layer and the suitable thin film of Al_2_O_3_ can strongly inhibit the electronic conductivity (Jung and Han, 2013). Additionally, to confirm the efficiency of this plasma enhanced method, the cycling performance of Al_2_O_3_@LNCM, synthesized form the normal solid state method, is also invested, as shown in Figure S5B. Compared to PLA-1-Al_2_O_3_@LNCM, 1-Al_2_O_3_@LNCM, reaches a discharge capacity of only 181.8 mAhŁEg^−1^ after 100 cycles, which only represents 92.38% of its initial discharge capacity (196.9 mAhŁEg^−1^). However, PLA-1-Al_2_O_3_@LNCM shows much higher capacity retention (98.46%) after 100 cycles, which further illustrates that the plasma-enhanced method has higher efficiency than that of the normal solid state method. These results clearly indicate that the materials obtained by the plasma-enhanced strategy can be used as a promising cathode material in lithium-ion batteries.

**Figure 6:**
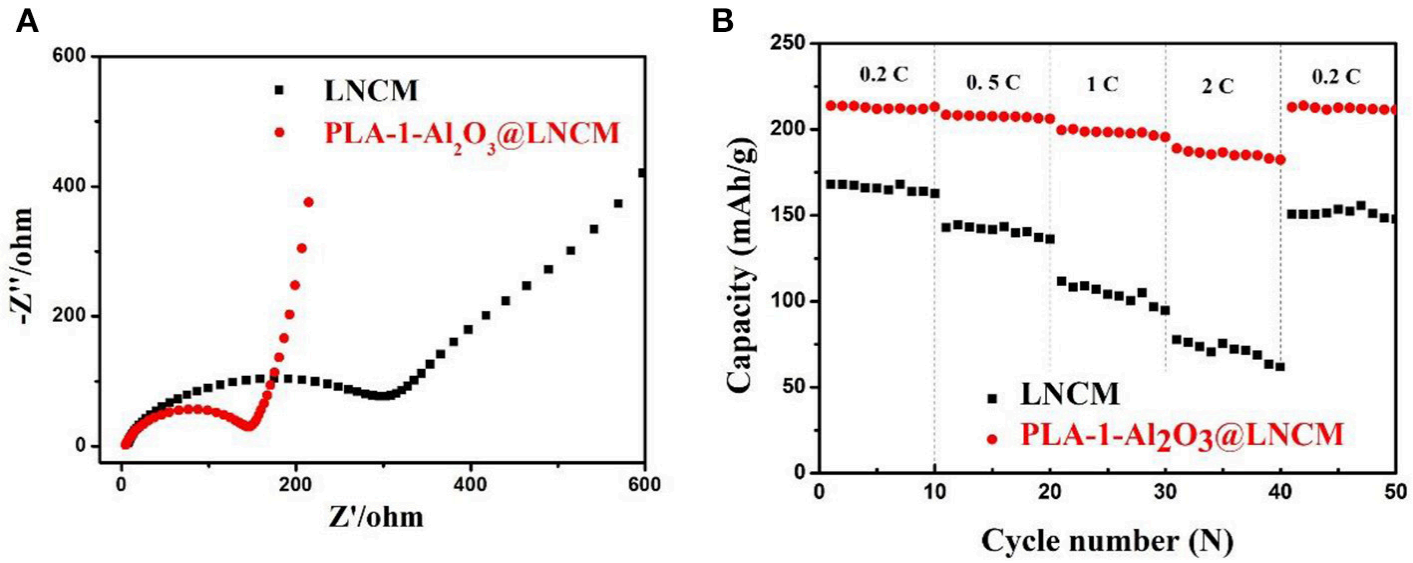
**(A)** Nyquist plots of bare LNCM and PLA-1-Al_2_O_3_@LNCM. **(B)** Rate capabilities vs. cycle number for bare LNCM and PLA-1-Al_2_O_3_@LNCM electrodes cycled at 0.2, 0.5, 1, 2, 0.2°C rates between voltage limits of 2.8 and 4.3 V.

To further investigate the effect of the Al_2_O_3_-coating on the electrochemical performance, the AC impedance curves of all the cycled samples were performed as shown in Figure 6A. The EIS plot consists of a depressed semi-circle in the high-medium frequency region that is attributed to the lithium-ion migration, through the SEI film and charge transfer reaction, and a straight line in the low frequency region attributed to the lithium-ion diffusion in the bulk electrode (Wang et al., 2014). A more detailed analysis of the impedance spectra was studied on the basis of the proposed equivalent circuit, where Rs represents the solution resistance and R_ct_ represents charge transfer resistance during the electrochemical reactions. The inclined line in the low-frequency region represents the Warburg impedance (Zw) associated with Li-ion diffusion in the electrode active material (Liu et al., 2004). EIS plots in Figure 6A were fitted using the equivalent circuit model. During cycling, the formation of the solid-state interface layer may lead to raised impedance. From the fitted impedance parameters, it can be seen that the charge transfer resistance (R_ct_) of PLA-1-Al_2_O_3_@LNCM is much smaller (R_ct_ ≈ 152.78 Ł) than that of the pristine LNCM (Rct ≈ 343.97 Ł), which indicates that the Al_2_O_3_ layer can enhance Li+ transportation with enlarged interlayer. Moreover, it may be due to the removal of the unidentified phase in the precycled electrode or the influence of a native passivation layer on the electrodes (Lin et al., 2014). Therefore, the decrease of impedance illustrates the effect of the Al_2_O_3_ layer on restraining the process during cycling, which further verifies the fact that the Al_2_O_3_ layer is responsible for the good performance of PLA-1-Al_2_O_3_@LNCM. In addition, the impedance reduction exhibits an enhancement in the kinetics of the lithium-ion diffusion through the surface layer, and an obvious increase in rate capability as shown in Figure 6B. During rate capability testing, the effect of the coating layer on improving capacity retention is relatively apparent. Rate measurement is performed as the currents gradually increase to 2°C, finally returning back to 0.2°C, as shown in Figure 6B. The pristine LNCM delivers a capacity of 168.2, 142.9, 111.8, and 77.8 mAhŁEg^−1^ at 0.2, 0.5, 1, and 2°C, respectively. For PLA-1-Al_2_O_3_@LNCM, the corresponding capacity is 213.8, 208.4, 199.6, and 189.0 mAhŁEg^−1^, respectively, which shows an obvious improved rate performance than that of the pristine LNCM. Moreover, when the current returns back to 0.2°C, the PLA-1-Al_2_O_3_@LNCM electrodes recover 99.58% of their original 0.2°C discharge capacity, compared to 89.66% for the uncoated electrode due to the attack from the electrolyte, further concluding that coating LNCM with Al_2_O_3_ can significantly improve its rate performance. The superior retention capability is due to the coating which improves the homogeneity of near-surface current density on the coated electrodes and uniform Al_2_O_3_ layer for the improvement transfer of lithium ions between the electrode and electrolyte. Therefore, the excellent rate performance can be attributed to the uniform coating, which can cut off contact with the electrolyte and suppress the dissolution of active substances.

**Figure 7:**
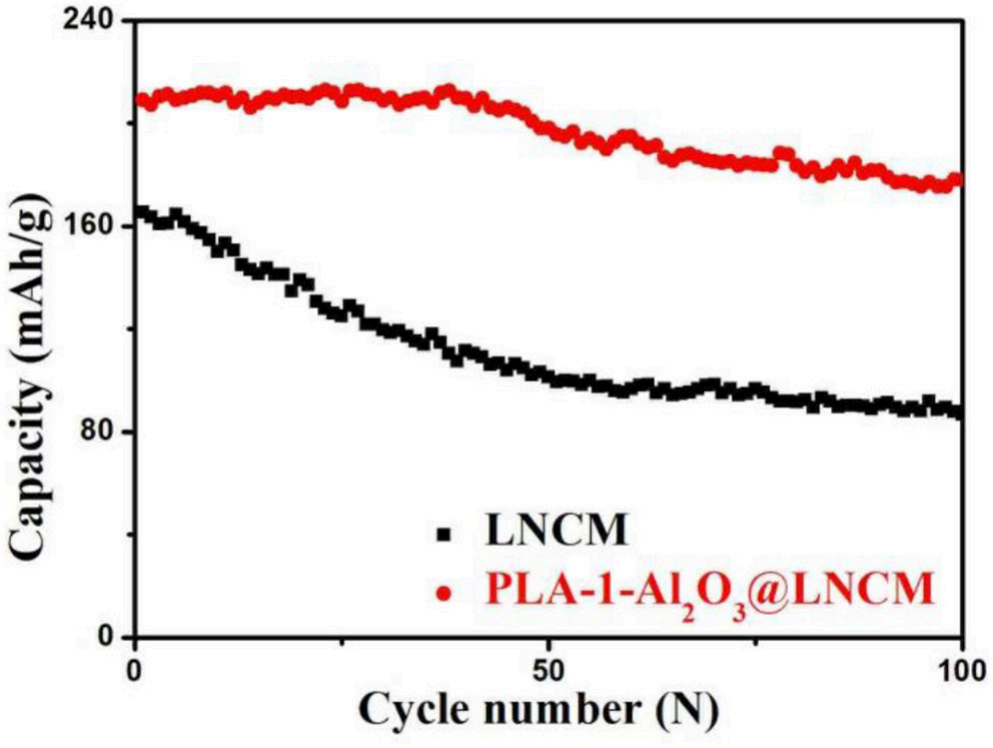
Cyclic performances at 0.2°C of LNCM and PLA-1-Al_2_O_3_@LNCM at 55°C.

The cyclic performances of bare LNCM and PLA-1-Al_2_O_3_@LNCM at 0.2°C in a potential range of 2.8–4.3 V at a high temperature (55°C) are shown in Figure 7. The initial discharge capacity of bare LNCM is 165.2 mAhŁEg^−1^, whereas that of the PLA-1-Al_2_O_3_@LNCM is 209.1 mAhŁEg^−1^. There is a lot of difference between the LNCM and PLA-1-Al_2_O_3_@LNCM sample, indicating that the 5 nm Al_2_O_3_ coating layer affects the Li+ diffusion between the LNCM particles and the electrolyte during the first cycle. More importantly, there are significant differences in cyclic performance between LNCM and PLA-1-Al_2_O_3_@LNCM as shown with the increasing cycling numbers. After 100 cycles, the discharge capacities of bare LNCM is only maintained at 86.6 mAhŁEg^−1^, suggesting that the material suffers stronger destruction as compared to the side reaction aggravated under a higher temperature. PLA-1-Al_2_O_3_@LNCM shows better capacity retention with 85.08% of the initial discharge, presumably to an inhibiting effect of the Al_2_O_3_ coating layer on the metal ion dissolution from the LNCM electrode to the electrolyte. In order to further confirm the effect of the plasma-enhanced method on the material, the cyclic performances of 1-Al_2_O_3_@LNCM are invested, as shown in Figure S6. The initial discharge capacity of PLA-1-Al_2_O_3_@LNCM is much higher than that of 1-Al_2_O_3_@LNCM, which indicates that the different synthesis methods have many differences on the first discharge cycle. Moreover, PLA-1-Al_2_O_3_@LNCM exhibited much better cyclic performance than that of 1-Al_2_O_3_@LNCM, which further confirms the superiority of the plasma-enhanced method in synthesizing the LNCM with the uniform Al_2_O_3_ layer. The proper coating using a special synthesis method can also suppress the formation of the passive film of the solid state electrolyte acting as a highly effective lithium ion conductor, which consequently improves the rate capability even at an elevated temperature. In addition, we can clearly see that PLA-1-Al_2_O_3_@LNCM exhibits excellent electrochemical performances compared to many relevant references as shown in Table S2.

## Conclusion

A novel and effective Al_2_O_3_ coating has been successfully achieved via a facile plasma-enhanced route method, investigating the effects of the Al_2_O_3_ coating on the high temperature cyclic performance of LNCM. From the physical test, it can clearly be seen that the Al_2_O_3_ uniformly grows on the surfaces of LNCM particles with a thickness of about 5 nm, causing no changes on the structure of LNCM. Futhermore, PLA-1-Al_2_O_3_@LNCM exhibited a better cyclic performance and its discharge capacity was 213.8 mAhŁEg^−1^ at 0.2°C, and its capacity retention was 98.46% after 100 cycles, which is much higher than that of bare LNCM and 1-Al_2_O_3_@LNCM synthesized using the normal solid state method. What's more, PLA-1-Al_2_O_3_@LNCM shows better rate capability and cycling stability, which can be attributed to the uniform coating of Al_2_O_3_. When cycling at 55°C, PLA-1-Al_2_O_3_@LNCM shows 85.08% capacity retention after 100 cycles, which is much higher than that of bare LNCM only, with a 52.42% capacity retention. The enhanced electrochemical performance further illustrates the efficiency and superiority of the Al_2_O_3_ layer, which is achieved using the plasma-enhanced method, and which can protect cathode material thus alleviating the severe dissolution of metal ions from dissolving in the electrolyte and reduces decomposition of the electrolyte at the cathode. This facile and efficient surface modification with low-cost starting material and an effective coating layer structure can be adopted to enhance the cycling performance and thermal stability for other electrode materials, which can be used to synthesize other candidate cathode materials for lithium ion batteries.

## Data Availability Statement

The datasets generated for this study are available on request to the corresponding author.

## Author Contributions

QJ and XW wrote and modified the manuscript. YZ tested the performances of materials. NY synthesized the materials. JT modified the manuscript.

### Conflict of Interest

The authors declare that the research was conducted in the absence of any commercial or financial relationships that could be construed as a potential conflict of interest.
