# Corrigendum: High Efficient and Environment Friendly Plasma-Enhanced Synthesis of Al_2_O_3_-Coated LiNi_1/3_Co_1/3_Mn_1/3_O_2_ With Excellent Electrochemical Performance

**DOI:** 10.3389/fchem.2020.596123

**Published:** 2020-10-16

**Authors:** Xinzhi Wang, Qianqian Jiang, Yichi Zhang, Nannan Yuan, Jianguo Tang

**Affiliations:** ^1^Institute of Hybrid Materials, National Center of International Research for Hybrid Materials Technology, National Base of International Science and Technology Cooperation, College of Materials Science and Engineering, Qingdao University, Qingdao, China; ^2^School of Chemical and Biomedical Engineering, Nanyang Technological University, Singapore, Singapore

**Keywords:** plasma-enhanced method, Al_2_O_3_ layer, LNCM, elevated temperature performance, Li-ion batteries

The author name order was incorrectly displayed as Qianqian Jiang^1,2^, Xinzhi Wang^1^, Yichi Zhang^1^, Nannan Yuan^1^ and Jianguo Tang^1^. The correct author name order is Xinzhi Wang^1^, Qianqian Jiang^1,2^, Yichi Zhang^1^, Nannan Yuan^1^ and Jianguo Tang^1^.

The authors apologize for this error and state that this does not change the scientific conclusions of the article in any way. The original article has been updated.

