# Supplementary material for: High Efficient and Environment Friendly Plasma-Enhanced Synthesis of Al2O3-Coated LiNi1/3Co1/3Mn1/3O2 With Excellent Electrochemical Performance
Source: Front Chem. 2020 Mar 20;8:72. doi: 10.3389/fchem.2020.00072 (PMC7099288; doi:10.3389/fchem.2020.00072)
Supplement: Supplementary file 1 [file Data_Sheet_1.docx]

**High Efficient and Environment Friendly Plasma-enhanced Synthesis of Al_2_O_3_-coated LiNi_1/3_Co_1/3_Mn_1/3_O_2_ with Excellent Electrochemical Performance**

Qianqian Jiang^a, b, #,*^, Xinzhi Wang^, #^, Yichi Zhang^a^, Nannan Yuan^a^, and Jianguo Tang^a, *^

^a^ Institute of Hybrid Materials, National Center of International Research for Hybrid Materials Technology, National Base of International Science & Technology Cooperation, College of Materials Science and Engineering, Qingdao University, Qingdao, 206000, P. R. China

^b^ School of Chemical and Biomedical Engineering, Nanyang Technological University, 62 Nanyang Drive, Singapore 637459, Singapore

^#^ These authors contributed equally to this work.

Correspondence to: Q. Q. Jiang (E-mail: [kaiqian2008@163.com](mailto:kaiqian2008@163.com) and jtang951@163.com)


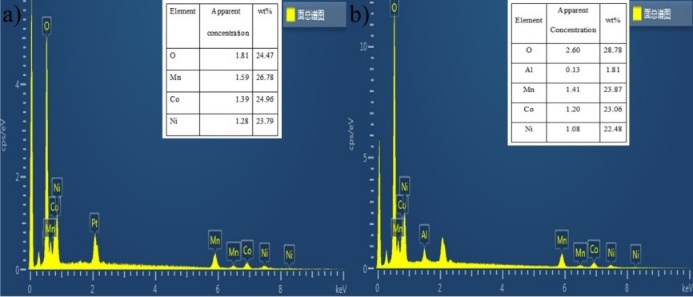


Figure S1. EDAX spectrum of (a) the pristine LNCM and (b) PLA-1-Al_2_O_3_@LNCM


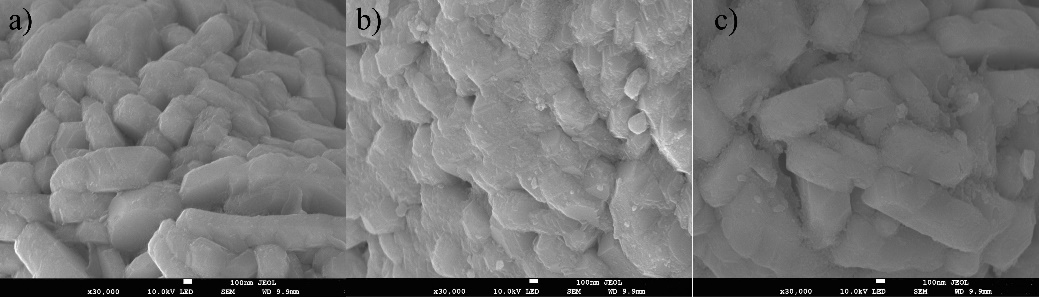


Figure S2. SEM photographs of PLA-Al_2_O_3_@LNCM with different Al consent: (a) 0.5 wt%, (b) 1 wt% and (c) 1.5 wt%


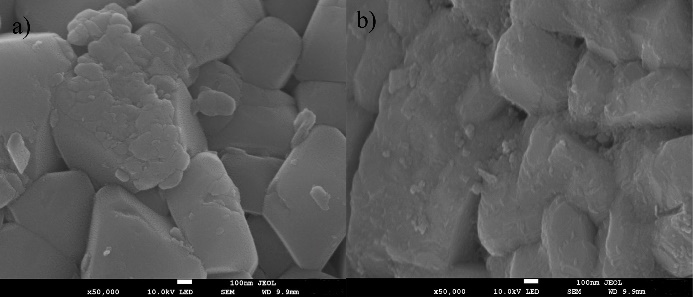


Figure S3. SEM images after 100 cycles of (a) LMCM and (b) PLA-Al_2_O_3_@LNCM.

Table S1 Lattice parameters of LNCM and LNCM-S-20

| Sample | a (Å3) | c (Å3) | c/a | I_003_/I_004_ | Li^+^/Ni^2+^ disorder | R_wp_ |
| --- | --- | --- | --- | --- | --- | --- |
| LNCM | 2.859 | 14.231 | 4.978 | 1.29 | 0.0467 | 8.38 |
| PLA-1-Al_2_O_3_@LNCM | 2.860 | 14.234 | 4.977 | 1.28 | 0.0385 | 7.68 |


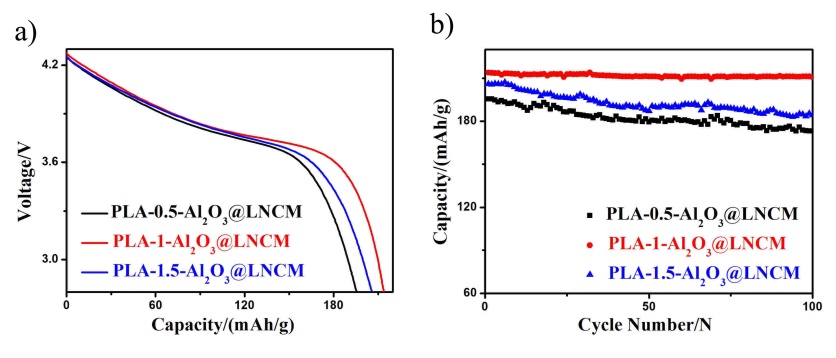


Figure S4. Electrochemical performance of Al_2_O_3_ coated LNCM with different Al consent: (a) Initial discharge, (b) Discharge capacities vs. cycle number


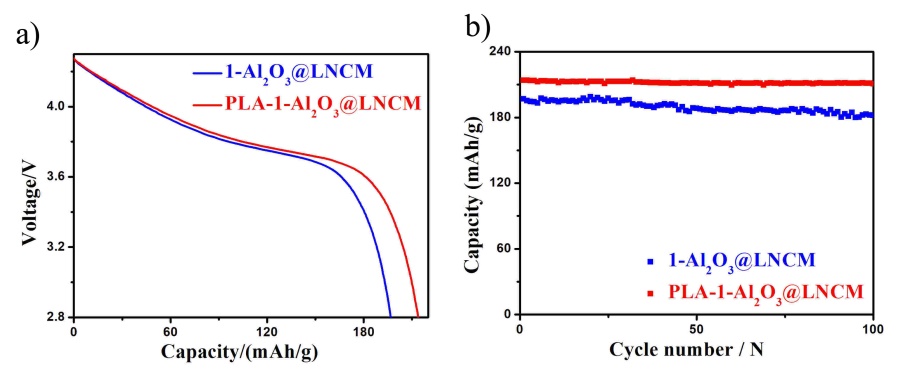


Figure S5. (a) Initial discharge and (b) Discharge capacities vs. cycle number of samples 1-Al_2_O_3_@LNCM and PLA-1-Al_2_O_3_@LNCM


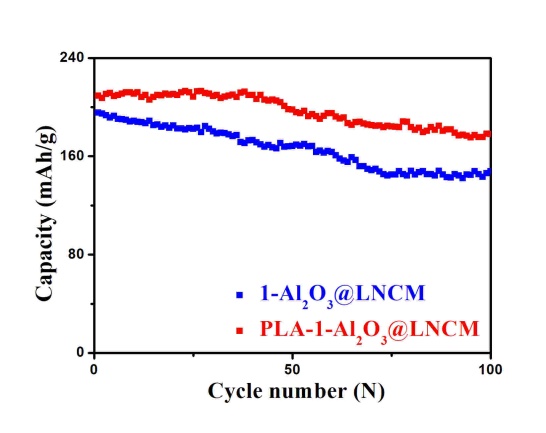


Figure S6. Cycling performances of 1-Al_2_O_3_@LNCM and PLA-1-Al_2_O_3_@LNCM at 55 ^°^C in the voltage range 2.8 - 4.3 V Li/Li^+^

Table S2. Representative electrochemical performance for LiNi_1/3_Co_1/3_Mn_1/3_O_2_ modified with different method from literature. *not mentioned

| Method | Modification Method | Initial Discharge  (mAh/g) | Cut off  condition（V） | Capacity  retention (%)  (0.2 C, 100 cycles) |
| --- | --- | --- | --- | --- |
| Plasma-enhanced Synthesis (This work) | Coated by uniform Al_2_O_3_ | 213.8 | 2.8 - 4.3 | 98.46 |
| Plasma-enhanced Method (Jiang et al. 2016) | Sulfur Atom-scale Modification | 200.4 | 2.8 - 4.3 | 94.46 |
| Sol–gel method (Qi et al. 2014) | Coated by Al_2_O_3_ | 172.8 | 3.0 - 4.5 | 97.5 |
| Simple combustion method (Cho and Ryu 2012) | Sulfur anion doping and surface modiﬁcation with LiNiPO_4_ | 200 | 2.8 - 4.3 | 92 (50 cycles) |
| A facile template method (Zhou et al. 2019) | Using single crystal β-MnO_2_ nanorods as self-template | 201.2 | 2.8 - 4.6 | 90.2 (200 cycles) |
| Urea homogeneous  precipitation method (Shen et al. 2015) | Coated by Al_2_O_3_ | 204.8 | 3.0 - 4.5 | * (0.5 C 90.3%) |
| Solid State Reaction with Zr(OC_4_H_9_) (Wang et al. 2015) | Li_2_ZrO_3_-coated on the surface | 200.4 | 3.0 - 4.6 | 89 |
| Solid State Reaction (Shao et al. 2015) | Coated by Al_2_O_3_ | about 156.3 | 2.8 - 4.6 | About 92.6 (20 cycles) |

**Reference**

Cho, S. W. and K. S. Ryu (2012) Sulfur anion doping and surface modification with LiNiPO4 of a LiNi0.5Mn0.3Co0.2O2 cathode. *Mater. Chem. Phys.,* 135**,** 533-540. doi: 10.1016/j.matchemphys.2012.05.021

Jiang, Q., N. Chen, D. Liu, S. Wang and H. Zhang (2016) Efficient plasma-enhanced method for layered LiNi1/3Co1/3Mn1/3O2 cathodes with sulfur atom-scale modification for superior-performance Li-ion batteries. *Nanoscale,* 8**,** 11234-11240. doi: 10.1039/c6nr02589g

Qi, Q., H. Xi, Y. Chen, T. Yan and W. Lv (2014) Al2O3 coated LiNi1/3Co1/3Mn1/3O2 cathode material by sol–gel method: Preparation and characterization. *Ceram. Int.,* 40**,** 10511-10516. doi: 10.1016/j.ceramint.2014.03.023

Shao, Z. C., J. Guo, Z. Zhao, J. Xia, M. Ma and Y. Zhang (2015) Preparation and Properties of Al2O3-doping LiNi1/3Co1/3Mn1/3O2 Cathode Materials. *Mater. Manuf. Processes,* 31**,** 1004-1008. doi: 10.1080/10426914.2015.1117618

Shen, D., D. Zhang, J. Wen, D. Chen, X. He, Y. Yao, X. Li and C. Duger (2015) LiNi1/3Co1/3Mn1/3O2 coated by Al2O3 from urea homogeneous precipitation method: improved Li storage performance and mechanism exploring. *J. Solid State Electrochem.,* 19**,** 1523-1533. doi: 10.1007/s10008-015-2740-z

Wang, W., Z. Yin, J. Wang, Z. Wang, X. Li and H. Guo (2015) Effect of heat-treatment on Li2ZrO3-coated LiNi1/3Co1/3Mn1/3O2 and its high voltage electrochemical performance. *J. Alloy. Compd.,* 651**,** 737-743. doi: /10.1016/j.jallcom.2015.08.114

Zhou, H., H. Cheng, H. Zhao, K. Zhao, Y. Zhao, J. Zhang, Q. Xu and X. Lu (2019) Superior Stability and Dynamic Performance of Single Crystal LiNi1/3Co1/3Mn1/3O2 Nanorods from β-MnO2 Template for Lithium-Ion Batteries. *J. Electrochem. Soc.,* 166**,** A59-A67. doi: 10.1149/2.0281902jes
